# Parental involvement in neonatal decision-making: a narrative review

**DOI:** 10.3389/fped.2026.1803025

**Published:** 2026-04-20

**Authors:** Yanhui Ma, Chongyang Zhang, Ling Lu, Mengyu Li, Xueni Tian, Junxiang Gao

**Affiliations:** 1 Department of Neonatology, The Second Hospital of Hebei Medical University, Shijiazhuang, Hebei, China; 2 Center for Medical Humanities Research, The Second Hospital of Hebei Medical University, Shijiazhuang, Hebei, China; 3 School of Nursing, Hebei Medical University, Shijiazhuang, Hebei, China; 4 Department of Discipline Inspection and Supervision Office, The Second Hospital of Hebei Medical University, Shijiazhuang, Hebei, China

**Keywords:** decision support, informed consent, neonatal intensive care, parental involvement, shared decision-making

## Abstract

In the neonatal intensive care unit (NICU), parents are frequently required to make preference-sensitive decisions under prognostic uncertainty and severe time pressure. Although family-centered care and shared decision-making (SDM) are widely advocated, evidence on how parental involvement varies across neonatal decision contexts remains fragmented. We searched multiple databases (MEDLINE, Embase, Web of Science, CINAHL, and PsycINFO) from inception to 5 September 2025 and included empirical studies reporting parental involvement in neonatal decision-making. Using a narrative synthesis, we organized and presented findings within a social–ecological framework (individual/family, clinical processes, team/organization, and policy/society). Across contexts, parental involvement is best understood as a dynamic continuum, ranging from clinician-led decision-making to SDM and, at times, parent-led decision-making. Most parents wish to be actively involved while also receiving clear clinical recommendations, and they need timely and comprehensible information, emotional support, and opportunities to revisit decisions as an infant's condition evolves. Greater perceived involvement is typically associated with better understanding, greater values–choice concordance, and lower decisional conflict and decisional regret. In contrast, routinized default pathways, inconsistent information across team members, and limited psychosocial support may undermine parental agency and increase burden. Actionable improvement targets include explicitly presenting options with clear framing, communicating uncertainty transparently, providing protected decision windows when feasible, and coordinating team roles to deliver consistent information.

## 1 Introduction

Advances in neonatal intensive care and perinatal medicine have substantially improved survival among critically ill newborns (1–3), while also increasing the number and complexity of decisions faced by clinical teams and families (4–6). Many of these decisions are preference-sensitive and occur under prognostic uncertainty, extreme time constraints, and intense emotional distress, including whether to initiate or withdraw life-sustaining treatment, how to escalate or limit invasive interventions, whether to pursue expanded newborn screening and genomic testing, and whether to participate in research (7, 8). Under such conditions, decision quality depends not only on clinical evidence, but also on how options and uncertainty are communicated, how roles and responsibilities are negotiated, and whether families receive practical support to participate in ways that align with their values.

International guidance from bodies such as the World Health Organization (WHO) and the Nuffield Council on Bioethics emphasizes family-centered care and recognizes parents as key partners in neonatal decision-making (9, 10). However, empirical studies repeatedly indicate a persistent gap between parents' desired level of involvement and the involvement achieved in routine practice. Reported barriers include insufficient or poorly timed information, limited opportunities to ask questions, inconsistent communication across team members, and consequent decisional conflict or decisional regret (4, 11, 12). Because the available evidence is often organized around single decision points or specific population subgroups, it can be difficult for researchers and practitioners to develop a comprehensive understanding of how parental involvement varies across contexts and which determinants are modifiable.

From a social–ecological perspective, neonatal intensive care unit (NICU) decision-making is shaped by multilevel, interacting influences, including individual and family characteristics, clinical communication processes, team and organizational structures, and broader policy and sociocultural norms (13). Upstream structural conditions (e.g., visitation policies, ward design, and information governance) may be “transmitted” through everyday workflows, thereby affecting parents' access to information, their opportunities to express preferences, and their perceptions of agency. Accordingly, this narrative review synthesizes qualitative and quantitative evidence on parental involvement across common neonatal decision contexts and maps determinants of involvement onto four social–ecological levels (individual/family, clinical processes, team/organization, and policy/society) to identify actionable leverage points for intervention. Our aims were to characterize patterns of involvement, summarize outcomes associated with involvement, and identify communication and workflow features that may strengthen high-quality parental involvement.

## 2 Methods

We systematically searched MEDLINE, Embase, Web of Science, CINAHL, and PsycINFO from inception to 5 September 2025 for English-language studies on parental involvement in neonatal decision-making. Search terms combined controlled vocabulary and free-text keywords, including parent*, parental involvement/participation, newborn*/neonat*, shared decision*, informed decision/choice, decision aid*, decision support, and decision making.

Records were imported into EndNote and deduplicated. Two reviewers independently screened titles and abstracts, followed by full-text assessment; disagreements were resolved through discussion, with adjudication by a third reviewer when necessary. We included peer-reviewed empirical studies (qualitative, quantitative, or mixed methods) conducted in neonatal care settings that reported parental involvement in decision-making and/or shared decision-making (SDM) processes. We excluded studies reporting clinician perspectives only without parental data; tool-development studies that did not report outcomes related to involvement/SDM; non-original publications (e.g., commentaries, editorials, conference abstracts, theses, and protocols); studies outside neonatal settings or with unclear age definitions; and reports without accessible full text.

**Figure 1:**
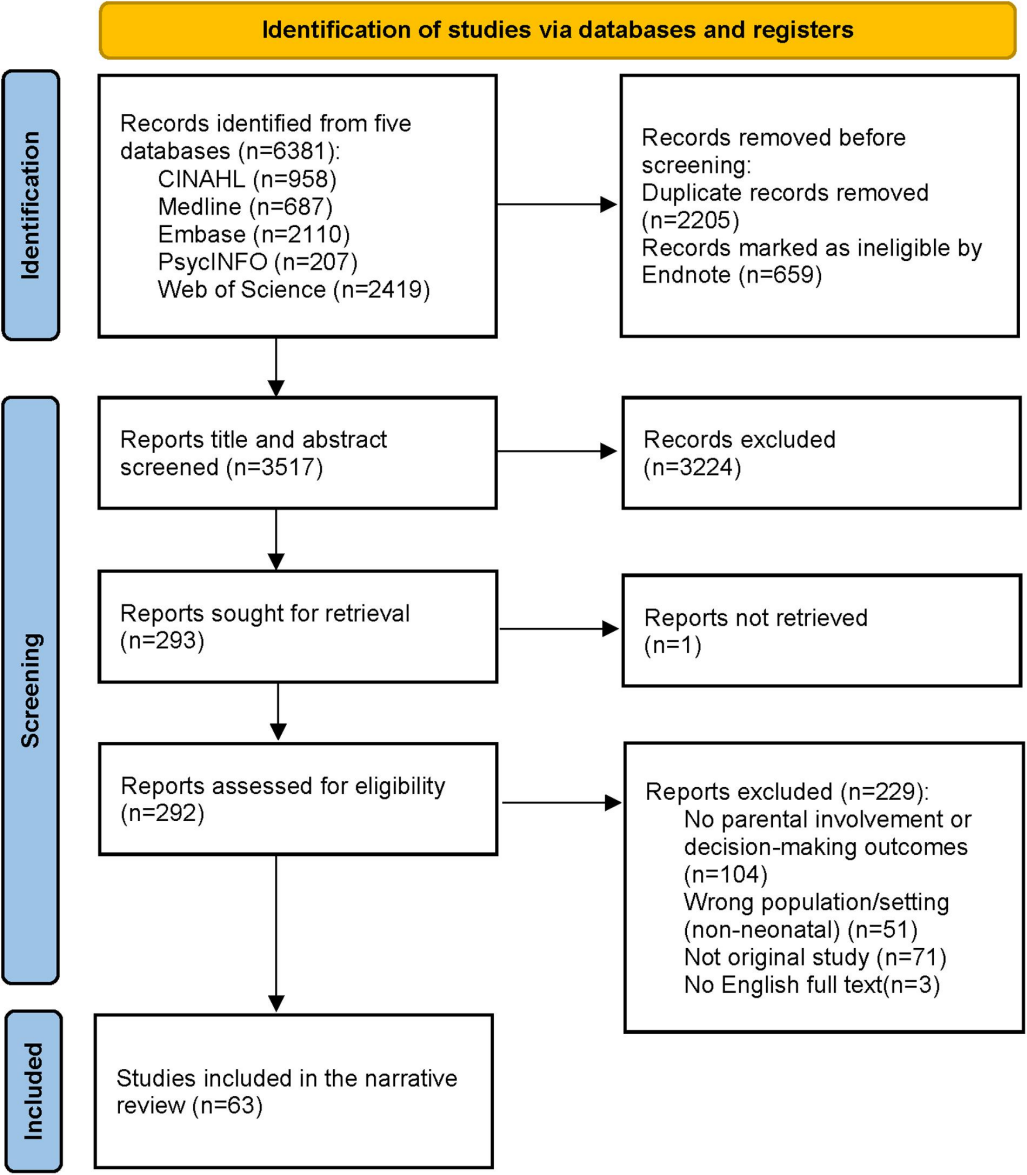
A flow chart for the data selection process.

Given substantial heterogeneity in decision types, study settings, and outcome measures, we did not perform a meta-analysis. Instead, we used a narrative synthesis to summarize the evidence. We then mapped determinants of parental involvement onto a social–ecological framework (individual/family, clinical processes, team/organization, and policy/society) to identify potentially modifiable factors. The search yielded 6,381 records, and 63 studies were included in the final synthesis (Figure 1). The included studies are summarised in Table 1.

**Table 1:** Overview of the included studies.

1st author	Country	Study design	Decision context	Participants	Data collection method(s)	Themes identified
Alderson (40)	UK	Qualitative	General NICU communication/participation: Parents’ information-sharing and decision participation with healthcare professionals (including informed consent and “everyday micro-decisions”).	80 parents	Field observation + semi-structured interviews	3.2;4.1;4.3;4.4;5.1;5.2;5.3;5.4
Axelin (51)	Finland	Qualitative	General NICU communication/participation: Neonatologist–parent communication patterns and level of involvement during ward rounds.	22 parents (8 mothers + 7 couples)	Video observation + interviews	4.1;4.2;4.3;5.3
Bailey (67)	USA	Quantitative	Screening/genomic testing (research recruitment): The impact of a decision-aid brochure on informed decision-making in Fragile × screening research recruitment.	1,323 parents	Questionnaire survey + interviews	5.2
Ballard (45)	USA	Quantitative	Research consent (consent validity): Understanding, recall, and voluntariness of perinatal trial consent (NEOPAIN).	64 parents	Open-ended questions + interviews	3.3
Banazadeh (23)	Iran	Qualitative	EOL limitation/withdrawal of treatment/palliative care: Factors influencing parental involvement in decision-making in the context of life-threatening conditions.	10 parents	Semi-structured interviews	3.1;3.2;4.3;4.4;5.1
Beyer (33)	Germany	Qualitative	EOL limitation/withdrawal of treatment/palliative care: Experiences and preferences regarding shared decision-making (SDM) in limitation/withdrawal decisions.	12 parents	Semi-structured interviews	3.2;4.1;4.2;4.3;5.1;5.2;5.3;6.2
Brinchmann (20)	Norway	Qualitative	EOL limitation/withdrawal of treatment/palliative care: Parents’ attitudes toward participation in life-and-death decisions for extremely preterm/critically ill neonates.	35 parents	Unstructured interviews	3.1;4.1;4.3;5.1;5.3
Burgess (47)	Canada	Quantitative	Research consent/trial recruitment: Experiences of NICU research recruitment and consent; attitudes toward “physicians making enrolment decisions on parents’ behalf.”	73 parents	Questionnaire survey	3.3;5.1;5.2;5.3
Caeymaex (18)	France	Qualitative	EOL limitation/withdrawal of treatment/bereavement: Narratives of parental roles in end-of-life decision-making and long-term emotional outcomes after neonatal death.	164 parents	Semi-structured interviews	3.1;3.2;4.1;4.2;4.3;5.1;5.3;6.2
Choi (61)	South Korea	Quantitative	General NICU communication/participation: Parents’ perceived SDM and its relationships with uncertainty and self-efficacy.	103 parents	Questionnaire survey	4.4;6.1
Currie (26)	USA	Qualitative	EOL limitation/withdrawal of treatment/palliative care: Parents’ experiences of hospitalisation, end-of-life care, and paediatric palliative care consultation (PPC).	10 parents (7 mothers + 3 fathers)	Semi-structured interviews	3.1;3.2;4.3;5.1;5.3;5.4
Einarsdóttir (19)	Iceland	Qualitative	EOL limitation/withdrawal of treatment/palliative care: Parents’ views on withdrawal criteria for ELBW infants and “who should decide.”	53 parents (28 mothers + 25 fathers)	Semi-structured interviews	3.1;3.2;3.3;4.1;4.2;4.3;5.1;5.2
Eisenhauer (42)	USA	Qualitative	Screening/biobank governance: Mothers’ decisions about donating residual newborn blood spots for research.	20 mothers	Field observation + semi-structured interviews	3.3;4.1;5.2
Esquerra-Zwiers (59)	USA	Qualitative	Feeding/breast milk (consent): Mothers’ decision-making process regarding consent for donor human milk (DHM) in the NICU.	20 mothers	Semi-structured interviews	4.1;5.1;5.3
Hendriks (17)	Switzerland	Qualitative	EOL limitation/withdrawal of treatment/palliative care: Parental involvement and decision satisfaction in end-of-life decisions for extremely preterm infants.	20 parents (7 couples + 5 mothers + 1 father)	Semi-structured interviews	3.1;3.2;4.1;4.3;5.1;5.3
Hoeben (69)	Netherlands	Quantitative	Team/organisation (FICare): Parents’ experiences of participating in care, rounds, and decision-making under FICare implementation.	344 parents	Online survey	5.3
Hoehn (64)	USA	Mixed-methods	Research consent/trial recruitment: Parents’ perceptions of whether there is “enough time to decide” about research participation (CHD context).	37 parents (18 fathers + 19 mothers)	Structured questionnaire + open-ended questions	5.1;5.2;5.3
Korotchikova (65)	Ireland	Mixed-methods	Research consent (non-therapeutic): Consent/decline experiences for an EEG study in healthy term newborns.	123 parents	Informal interviews + structured questionnaire	5.1;5.2
Leblond (39)	UK	Qualitative	Screening/genomic testing: Co-designed consent experience for newborn whole-genome sequencing screening (Generation Study).	105 parents	Semi-structured interviews	3.2;5.1;5.2;5.3
Lemmon (14)	USA	Qualitative	Prognostic communication/limit of viability: Prognostic communication and outcome trade-offs in extreme prematurity—differences between parents’ and clinicians’ perspectives.	16 parents (9 mothers + 7 fathers)	Semi-structured interviews	3.1;3.2;5.3
Limacher (34)	Switzerland	Qualitative	EOL limitation/withdrawal of treatment/palliative care: Communication and decision-making regarding LST vs. palliative care in life-limiting/life-threatening contexts.	8 parents	Interviews	3.2;4.2
Ma (72)	China	Quantitative	General NICU communication/participation: Decision dilemmas and SDM participation in treatment decisions for preterm infants (mechanisms: social support/health literacy).	225 parents	Questionnaire survey	6.1
McLeish (41)	UK	Qualitative	Research consent (simplified consent): Acceptability/feasibility of simplified “opt-out” consent in neonatal trials.	11 parents	Semi-structured interviews	3.2;5.1;5.2;5.3
Moody (58)	UK	Mixed-methods	Screening/genomic testing: Views on informed consent for expanded newborn screening (heel-prick).	171 parents	Focus groups + online survey	4.1;4.3;5.1;5.2;5.3;5.4
Morgan (66)	USA	Qualitative	Circumcision: Maternal circumcision decisions during postpartum hospitalisation, information sources, and knowledge gaps.	10 mothers	Semi-structured interviews	5.2;6.2
Moultrie (30)	USA	Qualitative	Screening/genomic testing: Parents’ views on neonatal NGS/genomic screening (for decision-support development, NC NEXUS).	66 parents	Interviews	3.2;3.3;5.1
Nassef (28)	Sweden	Qualitative	Acute treatment decisions: Parents’ experiences of therapeutic hypothermia (TH) for HIE in an FCC unit.	14 parents (7 mothers + 7 fathers)	Semi-structured interviews	3.2;5.1;5.2;5.3;5.4
Newcomb (56)	USA	Quantitative	Screening/biobank governance: Views on “opt-in” authorisation for a DBS biobank during postpartum hospitalisation.	465 mothers	Questionnaire survey	4.1;5.2;5.3
Nicholls (55)	UK	Qualitative	Screening (blood spots): How routinised pathways, timing of information, and presentation shape perceived “optionality/voluntariness.”	18 parents	Semi-structured interviews	4.1;5.1;5.2;5.3
Nicholls (57)	UK	Quantitative	Screening (blood spots): Parents’ perceptions of understanding, availability of choice, and informed choice.	154 parents	Questionnaire survey	4.1;5.1;5.3
Nicholls (37)	UK	Qualitative	Screening (blood spots): Factors influencing parents’ trade-offs and deliberation when accepting screening.	18 parents	Semi-structured interviews	3.2;4.1;5.1;5.2;5.3;5.4
Nicholls (71)	UK	Quantitative	Screening (blood spots): Determinants of decision quality when accepting NBS.	154 parents	Questionnaire survey	6.1
Orfali (22)	France, USA	Qualitative	General NICU communication/participation: Parents’ experiences of decision involvement, perceived autonomy, and clinician–parent relationships across NICUs with different cultures and policies (cross-cultural comparison).	75 mothers	Participant observation + semi-structured interviews	3.1;3.2;4.1;4.3;4.4;5.1;5.3;5.4;6.2
Özveren (70)	Turkey	Quantitative	Circumcision: Decision pathways, informed consent processes, and satisfaction.	623 parents	Online questionnaire survey	5.4;6.2
Palomaa (24)	Finland	Qualitative	Pain management: Factors influencing parental involvement in neonatal analgesia/comfort measures.	140 parents	Open-ended questions	3.1;4.1;4.4;5.1;5.2;5.3;5.4
Pant (54)	India, SriLanka, Bangladesh	Mixed-methods	Research consent (time-critical trial): Consent processes for the HELIX time-critical trial (LMIC).	314 parents (survey 294 + interviews 20)	Questionnaire survey + semi-structured interviews	4.1;5.3;6.2
Partridge (25)	Australia, Hong Kong (China), Japan, Malaysia, Taiwan, Singapore, USA	Mixed-methods	Prognostic communication/resuscitation: Counselling experiences and decision-maker preferences in delivery-room resuscitation/life-support decisions for VLBW infants.	327 parents	Face-to-face interviews + telephone interviews + questionnaire survey	3.1;4.1;4.2;5.1;5.4
Peay (36)	USA	Mixed-methods	Screening/genomic testing: Population DNA screening (Early Check): digital education plus e-consent to support informed decision-making.	1,847 patients (survey 1,823 + interviews 24)	Online survey + semi-structured interviews	3,2;3.3;5.1;5.2;5.3;6.1;6.2
Peng (53)	Taiwan	Quantitative	EOL limitation/withdrawal of treatment/culture: End-of-life decisions and cultural practices (DNR/withdrawal and family support).	50 parents	Case analysis	4.1;5.1
Penticuff (68)	USA	Quantitative	General NICU communication/participation: Effects of a workflow-embedded collaboration intervention on maternal uncertainty, decisional conflict, and perceived shared decision-making (SDM) in a VLBW population.	154 mothers	Questionnaire survey	5.2;6.2
Piette (62)	Belgium	Qualitative	EOL limitation/withdrawal of treatment/palliative care: Barriers and facilitators to parental participation in end-of-life decisions.	23 parents	Semi-structured interviews	5.1;5.2;5.3
Quinn (16)	USA	Qualitative	Palliative care (early): Early family-centred palliative care—decision-making, care planning, and coping.	16 parents (12 mothers + 4 fathers)	Semi-structured interviews	3.1;3.2;4.1;4.2;4.4;5.1;5.2;5.3
Ranchod (49)	South Africa	Mixed-methods	Resource-constrained context: Parents’ role experiences in perinatal counselling and VLBW life-support decisions in public hospitals.	51 parents	Questionnaire survey + interviews	4.1;4.2;4.3;5.3
Richards (43)	UK	Qualitative	Research consent/multi-study recruitment: Parents’ experiences of concurrent recruitment (co-enrolment) for multiple studies involving preterm infants.	17 parents	Semi-structured interviews	3.3;5.1;5.2;6.2
Rohmah (74)	Indonesia	Quantitative	Feeding/breast milk: Breastfeeding decision-making during hospitalisation—pathway of family support → communication → SDM.	200 mothers	Questionnaire survey	6.1
Rohmah (73)	Indonesia	Quantitative	General NICU communication/participation: Mechanisms by which knowledge/trust/interaction influence SDM in routine care decisions.	92 parents	Questionnaire survey	6.1
Sagaser (63)	USA	Mixed-methods	Acute treatment decisions: Experiences of HIE/TH treatment—communication quality, perceived involvement, and implications for trauma-informed care.	165 parents	Online survey + open-ended questions	5.1;5.2;5.3
Saint (32)	France	Qualitative	EOL limitation/withdrawal of treatment/palliative care: Parental meaning-making and coping in limitation/withdrawal decisions.	12 parents (7 mothers + 5 fathers)	Semi-structured interviews	3.2;4.1;4.3;5.3
Schouten (31)	Germany	Qualitative	EOL limitation/withdrawal of treatment/palliative care: Extent of SDM implementation in end-of-life conversations (real-life conversation analysis).	23 parents	Interviews	3.2;5.1;5.2
Shah (44)	USA	Quantitative	Research consent/trial recruitment: PENUT recruitment—consent timing/identity of the consenter and decisional conflict.	163 parents	Questionnaire survey	3.3;5.2;6.1
Shaw (52)	UK	Qualitative	EOL limitation/withdrawal of treatment/palliative care: Decision conversations and initiation when shifting from intensive treatment to palliative care.	31 parents	Interviews	4.1;4.2;5.2
Soltys (60)	USA	Quantitative	General NICU communication/participation: Decision involvement and decisional regret after NICU hospitalisation experience.	400 parents	Online survey	4.2;6.1
Timmins (35)	USA	Qualitative	Screening/genomic testing: Parents’ views on expanded genomic screening (ethics/privacy and educational needs).	35 mothers	Semi-structured interviews	3.2;3.3;4.1;5.1;5.2
Ursin (15)	Norway	Qualitative	Periviability counselling: Whether parents should decide life-saving treatment at the limit of viability—roles and burden.	12 parents	Semi-structured interviews	3.1;3.2;4.1;4.2;5.1;5.4
Uveges (29)	USA	Mixed-methods	Major congenital anomalies: “Good parent” decision-making beliefs—differences between prenatal vs. postnatal diagnosis.	60 parents (survey 40 + interviews 20)	Field survey + semi-structured interviews	3.2;4.4;5.1;5.3
van der Pal (38)	Netherlands	Quantitative	Screening (blood spots): Parents’ views on accepting/declining/expanding NBS.	853 parents	Questionnaire survey	3.2;5.1;5.2;6.2
Verhoeven (21)	Netherlands	Mixed-methods	EOL limitation/withdrawal of treatment/palliative care (high-risk surgery): Surgical NEC—decision profiles/framework for surgery vs. palliative care.	53 parents	Q methodology	3.1;3.2;4.2;5.1
Weiss (7)	USA	Qualitative	General NICU communication/participation: How decision characteristics shape parents’ control preferences (delegating vs. retaining control).	30 parents	Semi-structured interviews	4.4
Weiss (8)	USA	Quantitative	General NICU communication/participation: Predictors of “parent-centred vs. medical team–centred” preferences across NICU decisions.	136 parents	Questionnaire survey	4.4
Weiss (48)	USA	Quantitative	Research consent/trial recruitment: HEAL trial—recruitment experiences, differences between consenters/decliners, and decisional conflict.	269 parents	Questionnaire survey	3.3;5.2;6.1
Wocial (50)	USA	Qualitative	EOL limitation/withdrawal of treatment/palliative care: Parents’ experiences of withholding/withdrawing life-sustaining treatment in the NICU.	20 parents	Open-ended interviews	4.1;5.2;5.3
Xu (27)	Australia	Quantitative	Circumcision: Parents’ motivations, attitudes, and information needs regarding circumcision (Victoria).	136 parents（62 fathers + 74 mothers）	Questionnaire survey	3.1;5.3;5.4
Zupancic (46)	Canada	Quantitative	Research consent/trial recruitment: Determinants of authorising clinical trial participation (risk–benefit, attitudes, and “preference for clinician recommendation”).	140 parents	Questionnaire survey	3.3;4.1

## 3 Core motivations for parental involvement in decision-making

Across an infant's illness trajectory, parental involvement in neonatal decision-making is primarily driven by three recurrent yet context-sensitive motivations: a preservation instinct during acute crises, a “best interests” calculus as the clinical course stabilizes, and altruism in research contexts.

### 3.1 Preservation instinct and parent–infant attachment

In early acute decision-making situations in the NICU, decision windows are often extremely narrow and uncertainty is high (e.g., during emergent airway management, hemodynamic instability, or the need for rapid escalation of life-sustaining support). Under time pressure—when prognosis is difficult to judge and there is little opportunity for careful deliberation—parental involvement frequently adopts a preservation-oriented stance: parents seek to avoid “missing a chance” and tend to endorse initiating or escalating available life-sustaining interventions (14–22). Importantly, this is not a universal pattern. Even in the acute phase, parental goals may lie along a continuum from maximizing survival to prioritizing comfort and relief of suffering (including setting limits on treatment, forgoing further escalation, or transitioning to comfort-focused care), and these goals may be dynamically revised as clinical information accumulates, the illness trajectory evolves, and parents' understanding of benefit–burden trade-offs deepens. Under these conditions, parents may place greater weight on the moral imperative to “do something” than on probabilistic trade-offs, and detailed discussions of long-term outcomes are often difficult to fully process in the moment. Prior reproductive experiences (e.g., infertility treatment, previous loss) and perceptions of the current pregnancy or infant as “unique” may further amplify a preservation orientation, making it emotionally and morally harder to accept limits on escalation or withdrawal (23).

Importantly, a preservation orientation often persists beyond the acute crisis, but the weighting of outcomes shifts as the clinical course stabilizes—away from “survival at all costs” and toward protecting the infant's day-to-day experience, including pain, procedural burden, and comfort. Parent–infant contact (e.g., kangaroo skin-to-skin care, feeding, soothing) appears to operate both as an attachment mechanism and as a pathway to engagement: hands-on caregiving can strengthen parents' sense of role legitimacy and self-efficacy, which in turn supports more active expression of preferences for analgesia, comfort-focused care, and avoidance of non-beneficial suffering (16, 24–26). A similar emphasis on experiential outcomes (especially pain) is also evident in decisions that are not explicitly life-and-death, where such outcomes may dominate parents’ trade-offs. For example, among parents who presented requesting neonatal circumcision, approximately 79.4% reportedly identified pain as their primary concern (27). This suggests that, even when parents ultimately proceed with the procedure, anticipated pain may remain a central consideration and shape their appraisal of the procedure's risks and benefits.

### 3.2 Best interests

As infants transition from an acute, highly time-pressured phase to a more stable or chronic trajectory, parents' reasoning more often shifts toward a “best interests” framework. The literature generally does not treat best interests as a single endpoint (e.g., survival), but rather as a multi-attribute calculus in which parents simultaneously weigh long-term survival, neurodevelopmental and functional outcomes, expected quality of life, and immediate comfort (including pain and the burden of invasive interventions) (14, 18, 19, 21–23, 28–34). Crucially, these trade-offs are embedded within family life: caregiving capacity, financial resources, family structure, and the needs of other children are commonly incorporated into judgments about what constitutes an acceptable and sustainable trajectory (15, 17, 18, 22, 34). Accordingly, different choices under similar prognostic estimates may reflect differences in acceptability thresholds rather than inadequate understanding. In high-burden contexts such as limiting or withdrawing life-sustaining treatment, moral language around “giving up” can activate anticipated regret and self-blame, leading some families to favor maximal treatment intensity to avoid the feeling that they “did not do everything possible” at the time (15, 26). However, multiple studies also show that many parents do not equate best interests with survival at any cost; they attach comparable importance to analgesia, reducing procedural burden, supportive touch, and continuity in relationships with the clinical team (16, 17, 19, 22, 28, 29, 31–39). This suggests that communication should not focus solely on metrics such as survival rates, but should also operationalize experiential outcomes—comfort, suffering, and dignity—with equal specificity and clarify how these dimensions enter the trade-off.

In screening and genomic testing, parents often operationalize best interests through “actionability”: they are more willing to test early when results can clearly guide interventions or management pathways, whereas high uncertainty and limited actionable options may prompt deferral or constrained disclosure to reduce anxiety and informational burden (30, 35–41). Supporting high-quality decisions within a best-interests framework therefore requires more than risk communication; it also requires explicit presentation of the full outcome set that matters to families and a clear account of what additional information can—and cannot—enable in terms of action.

### 3.3 Altruism

In research recruitment and biospecimen governance (including secondary use of residual samples), altruism is a recurrent, cross-context motivation. Qualitative and quantitative studies indicate that parents often describe participation as a way to “help future babies” and advance medical progress (19, 30, 35, 36, 42–48). A consistent explanatory thread is intergenerational reciprocity: parents view current medical advances as cumulative contributions from prior participants and construe their own participation as repayment and continuation of a public good (19, 43). Quantitative evidence further suggests that altruism influences decision-making for approximately 94% of parents, with higher endorsement among those who consent (98%) and lower among those who decline (84%) (46). Altruism, however, frequently co-exists with expectations of individual- or relationship-level benefits (e.g., increased attention, follow-up, or access to information), particularly when perceived risk is low (30, 36, 42–45, 47, 48). This “mixed-motivation” structure is not inherently problematic, but it may increase the risk of therapeutic misconception. High-quality consent should clearly distinguish research aims from direct clinical benefit, transparently discuss risks and uncertainty, and affirm parents' altruistic intentions in a respectful manner without overstating personal benefit.

## 4 Decision-making patterns and control preferences

**Figure 2:**
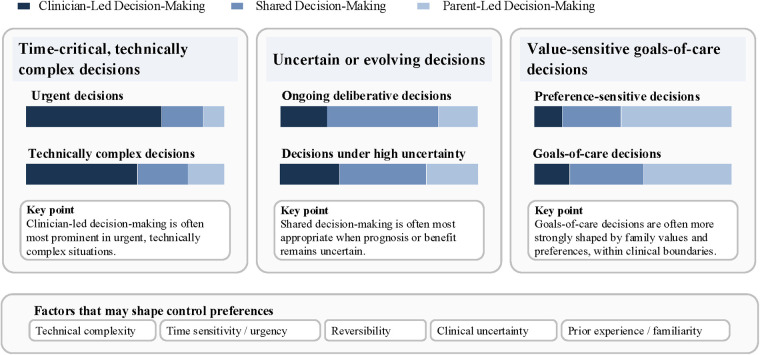
Common decision-making patterns across neonatal clinical contexts. The figure illustrates common patterns in the relative influence of clinicians and parents in decision-making across neonatal clinical contexts. The three panels represent time-critical, technically complex decisions; uncertain or evolving decisions; and value-sensitive goals-of-care decisions. Within each panel, the colored bars indicate the relative weight of clinician-led decision-making, shared decision-making, and parent-led decision-making. The boxed statements summarize key features of decision-making within each context, and the box below highlights factors that may shape control preferences.

In this review, “decision-making patterns” refer to the interactive operating mode through which options are generated and responsibilities are allocated between clinicians and parents; “control preferences” describe the extent of decision authority and moral responsibility that parents wish to assume in a given context (Figure 2). These patterns are better understood as different configurations that emerge across clinical contexts, comprising clinician-led decision-making, shared decision-making (SDM), and, in some situations, parent-led decision-making. The pattern that ultimately emerges in a given situation appears to be shaped jointly by the clinical context, decision characteristics, how options and uncertainty are communicated, and parents' perceptions of their own capacity to participate.

### 4.1 Clinician-led decision-making

Clinician-led decision-making is most common in highly acute, technically complex NICU situations with extremely narrow time windows (e.g., emergent airway management, hemodynamic instability, or rapid escalation of life-sustaining support). Clinicians typically lead immediate stabilization to prevent imminent harm, often guided by time-sensitive protocols. Subsequent inflection points (e.g., further escalation, treatment ceilings, or transition to comfort-focused care) require timely communication with parents and explicit goal alignment. When full deliberation is not feasible, parents are often included through rapid updates and brief, permission-seeking exchanges (16–18, 32, 33, 49, 50). Cross-national evidence suggests substantial variation in how acceptable families find “doctors deciding alone,” and clinician leadership is not uniformly interpreted as either “protection” or “exclusion” (25).

From an interactional perspective, clinician-led care often involves parental assent communicated through nodding, silence, or brief replies, with fewer invitations to participate in prospective planning (16, 17, 22, 51, 52). This does not necessarily indicate passivity: some families, under high stress or in highly technical situations, prefer to be informed and listened to rather than to bear ultimate decision authority (16, 20, 22, 50, 51). Accordingly, quality should not be judged by whether parents “made the decision,” but by whether the team transparently explains the rationale for recommendations, clarifies where genuine discretion exists, and preserves opportunities for questions and values expression. Evidence suggests two pathways to clinician-led decision-making (15, 19, 22–25, 33, 35, 40, 46, 51, 53, 54). The first is deliberate delegation by parents under emotional overload, high uncertainty, or perceived low health literacy; however, even when parents delegate, clinicians should actively elicit and document parental values and goals to guide subsequent decisions. The second is routinization embedded in workflows, whereby preference-sensitive choices become experientially “non-optional” through default pathways. This is particularly evident for routine procedures such as newborn bloodspot screening, where parents sometimes report that consent was not explicitly sought and screening was framed as standard practice (37, 55, 56). For example, a United Kingdom (UK) study found that although 70.1% of parents believed they had made an informed choice, 79.8% described screening as routine; 39.6% did not view it as “optional,” and 31.8% did not feel they could refuse at the time (57). A UK mixed-methods study reported similar findings (58). Notably, default acceptance does not necessarily imply coercion (42, 59). In the immediate aftermath of a crisis, clinician leadership may be protective by temporarily absorbing moral burden (20, 33). However, if strong clinician leadership persists beyond the acute phase due to routinization, it may undermine parental agency and increase subsequent distress and regret (15, 33). These findings underscore the need, once the clinical course stabilizes, to establish explicit “renegotiation points” (e.g., structured debriefs/reviews, renewed invitations for parental input, and clear presentation of value-sensitive trade-offs) to prevent “time-critical support” from hardening into a long-term constraint on partnership.

### 4.2 Shared decision-making

When an infant's condition is relatively stable, more than one medically reasonable option exists, and time allows deliberation, SDM is most feasible. Its core is not information quantity, but whether clinicians can translate the option set—benefits, burdens, and uncertainty—into a comparable structure and actively elicit parents' goals, concerns, and acceptability thresholds, so that parents experience their values as substantively shaping the plan (19, 21, 33, 34, 51, 52). Parents commonly regard being listened to and having their judgment taken seriously as hallmarks of SDM quality; even when the clinical course is difficult or the outcome is not as parents had hoped, those who perceive a shared, careful deliberation may find it easier to accept events and process emotions (15, 16, 18, 33, 51). Quantitative studies likewise show that parents endorse SDM more strongly than models in which clinicians decide alone (18, 25, 60). However, the literature also highlights substantial implementation gaps. In a South African survey of parents of extremely low birth weight infants, 49% ideally preferred to share decisions with clinicians, yet only 14% retrospectively reported that SDM actually occurred (49). This discrepancy likely reflects not only limitations in decision-making processes, but also broader structural and contextual constraints in the study setting, including resource constraints and inequities in access to care, socioeconomic barriers, and institutional, linguistic, and cultural/ethnic communication challenges. Accordingly, reproducible SDM processes may be necessary, but are unlikely to be sufficient unless they are implemented with attention to the realities of the clinical setting. SDM should be embedded as a workflow “bundle”: explicit option framing and boundary-setting, transparent communication of uncertainty, protected time for questions, clear within-team roles, and accessible written/video decision-support materials paired with follow-up conversations.

### 4.3 Parent-led decision-making

Parent-led decision-making most often arises in contexts characterized by high uncertainty and high moral burden, particularly trade-offs regarding limiting or withdrawing life-sustaining treatment. Conceptually, “parent-led” should not be understood as clinician withdrawal or permissiveness; rather, it denotes that, within a medically defensible set of options, families experience a real choice and make an informed, values-concordant decision (17, 19, 26, 33, 40, 58). Studies indicate that parent-led decisions are rarely made in complete independence; more commonly, they evolve through iterative conversations in which teams progressively synthesize evidence and prognostic judgments to narrow the option set (17, 18, 22, 32, 40, 51). If “consent” is used procedurally to shift responsibility, parents may experience being “pushed to the front line,” increasing traumatic burden (18, 40). Many parents want clinicians to state a clear professional position and explicitly share ethical responsibility to reduce the imprint of “sole responsibility” (19, 20, 22, 32, 40, 49). Parent-led decisions may also be more likely when families judge proposed interventions to be ineffective or futile, or when continued treatment is perceived as driven more by legal/institutional constraints than by expected benefit (23, 40). Conversely, when the final plan aligns clearly with core family values (e.g., avoiding non-beneficial suffering and preserving dignity), parents more often integrate the process as meaning-making and self-affirmation (18). Accordingly, teams should delineate the medically appropriate option set and the boundaries of uncertainty, make value-sensitive trade-offs explicit, and prevent “parent-led” models from degenerating into unilateral transfer of moral burden by clearly articulating shared responsibility and maintaining accessible opportunities for revisiting decisions.

### 4.4 Control preferences

Evidence suggests that parental control preferences are more state-like and context-dependent than stable traits. Technical complexity, time pressure, perceived reversibility, risk perception, and parents' prior experience or familiarity with a given decision appear to predict desired control more strongly than demographic factors (7, 8). In highly technical, time-critical interventions (e.g., endotracheal intubation, central venous catheter placement, emergency surgery), parents are more likely to delegate immediate procedural decisions to clinicians (7, 8, 24, 40). Nevertheless, escalation may still be limited by parent-endorsed goals of care, including DNR/DNI, treatment ceilings, or comfort-focused pathways. In lower-risk decisions that more closely resemble everyday parenting (e.g., feeding, kangaroo skin-to-skin care, participation in minimal-risk research), parents more often prefer greater control and view such decisions as extensions of the parenting role (7, 8, 16, 22, 29, 40). For moderate-risk, process-driven decisions, preferences are particularly sensitive to the communication environment: clear recommendations can increase acceptance of clinician leadership, whereas higher risk or uncertain benefit more often prompts parents to delay, retain veto power, and seek additional opinions (7). Individual psychological factors further modulate these patterns: under high uncertainty and low self-efficacy, parents may be more likely to relinquish control, whereas higher self-efficacy is associated with stronger perceived SDM (23, 24, 61). These findings support treating control preferences as an intervention target: reducing terminology and cognitive load, clarifying sources of uncertainty, and providing tiered information that can be used for trade-offs; creating opportunities for follow-up discussions and repeatedly “re-inviting” parental involvement, thereby improving perceived control and participation quality without imposing disproportionate moral burden.

## 5 Factors influencing parental involvement in decision-making

**Figure 3:**
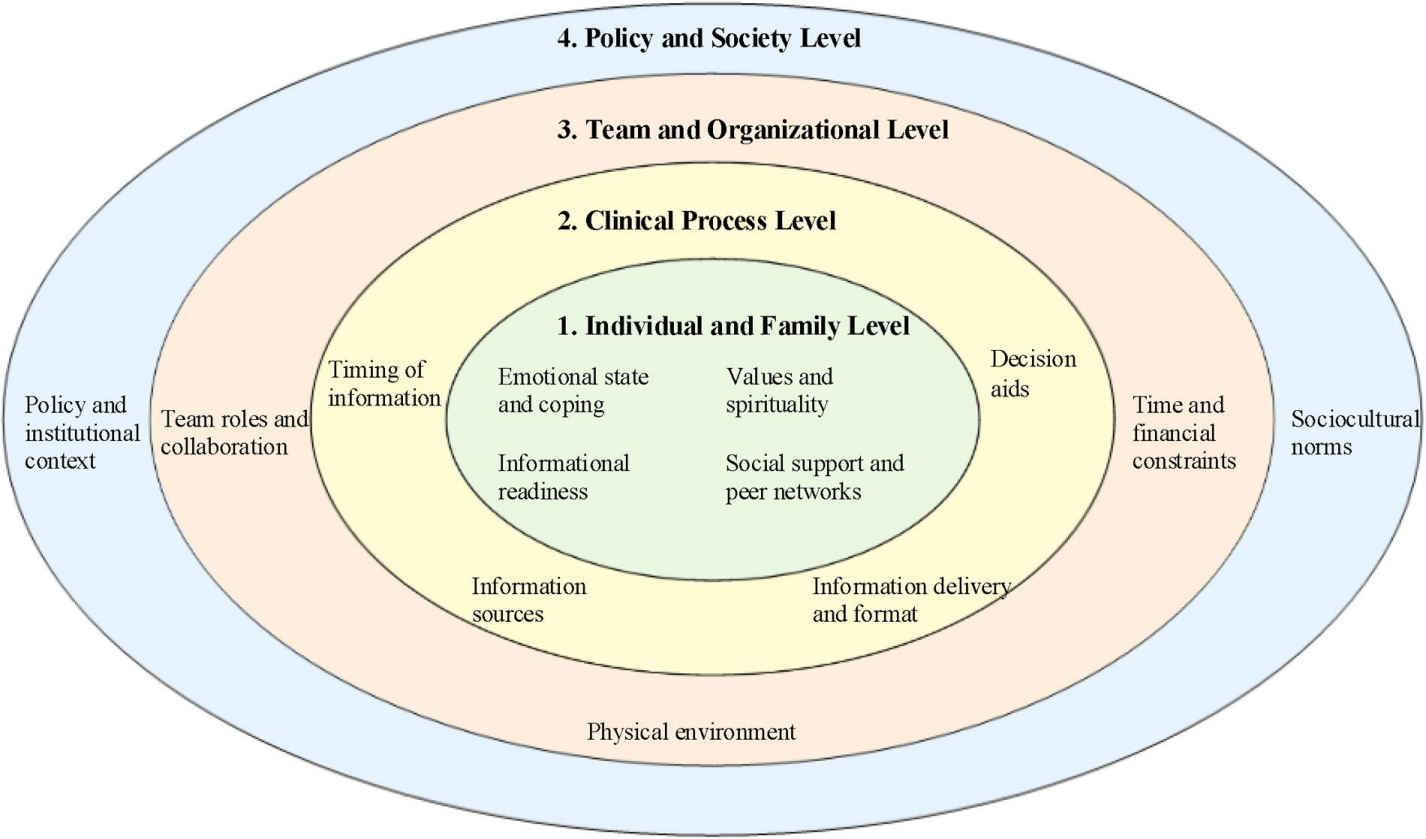
Socioecological multilevel framework of parental involvement in neonatal decision-making. Concentric circles from the centre outwards represent the individual and family, clinical process, team and organisational, and policy and societal levels. Together, these levels illustrate how multilevel factors shape parents’ opportunities for involvement and their experiences of participation in neonatal decision-making.

Parental involvement in neonatal decision-making is not determined by “willingness” alone. Rather, it emerges from interacting determinants operating across multiple levels of the care ecosystem. Using a social–ecological framework, we organized these determinants into four interdependent levels—individual- and family-level factors, clinical process-level factors, team- and organization-level factors, and policy- and society-level factors (Figure 3).

### 5.1 Individual- and family-level factors

At the individual and family level, parental involvement is shaped primarily by parents' changing ability to cope with uncertainty, including information processing, emotional regulation, and readiness to assume (or decline) moral responsibility. Across studies, interrelated determinants can be summarized into four domains: emotional state and coping, informational readiness (including health literacy), values and spirituality, and social support and peer networks.

#### 5.1.1 Emotional state and coping

Early in the NICU trajectory, abrupt clinical deterioration and time-critical interventions can trigger acute stress and hypervigilance, increase cognitive load, and disrupt attention allocation and information integration, thereby reducing the capacity for systematic deliberation and option comparison (15–18, 20, 33, 55, 62). Even parents with medical training may struggle to sustain stable risk–benefit trade-offs under high uncertainty and escalating informational demands (18, 22, 33). Several studies describe parents of extremely preterm infants feeling that information “arrives like a flood” in the first days after admission, supporting the view that consent and decision-making should be treated as an iterative process revisited at key junctures rather than a one-time event (41). During this period, grief, fear, guilt, and distress related to the infant's pain are often associated with avoidance-oriented coping and lower involvement in high-stakes or value-sensitive choices (16–18, 21, 23, 24, 40, 62). Quantitative data also suggest that emotional distress can function as a “pause point”; for example, approximately 9.7% of parents reported being unable to decide immediately about newborn bloodspot screening due to emotional distress (57). Families also differ in emotion regulation and information processing styles. Asynchrony between partners is common—for instance, one parent may prefer analytic reasoning while the other relies more on affective processing (62). Such asynchrony may amplify preference differences, complicate communication with clinicians, and increase the difficulty of forming a shared position over time.

#### 5.1.2 Informational readiness

Across contexts, a major constraint on substantive involvement is often insufficient informational readiness rather than lack of willingness to participate (15, 16, 22, 24, 26, 28, 33, 35, 36, 38, 50, 58). In one bloodspot screening study, approximately 83% of parents who completed screening were classified as having made an informed choice and about 86% demonstrated adequate knowledge; among those who declined, only about 44% met criteria for an “informed refusal,” and approximately 62% demonstrated adequate knowledge (38). Parents repeatedly emphasize that information should be complete, comprehensible, timely, and actionable, and often prefer tiered explanations that connect everyday language with technical terms. They also value consistency across team members and formal feedback of results, including negative results (25, 35–37, 39, 40, 43, 47, 50, 55, 58, 62, 63). These findings indicate that the critical outcome is not simply whether information is provided, but whether it is usable for deliberation.

#### 5.1.3 Values and spirituality

Under high uncertainty, values and religious/spiritual resources may function as coping and meaning-making mechanisms, supporting trust and willingness to collaborate with clinicians (19, 22, 23, 26, 29, 40). In end-of-life contexts, rituals may facilitate grief processing (26, 31, 33, 53). At the same time, when doctrinal requirements conflict with clinical judgment, religious commitment may create non-negotiable constraints, contributing to tension and delays (18, 23, 30, 59). Early involvement of chaplaincy/spiritual care and non-judgmental values clarification may help transform value differences into discussable inputs rather than adversarial impasses (19).

#### 5.1.4 Social support and peer networks

Involvement is also shaped by feasibility—whether parents can be present and whether decisional responsibility can be shared. The presence of both parents is often associated with greater perceived control and higher decision concordance; conversely, work demands, caregiving responsibilities for other children, and visitation restrictions can increase cognitive and emotional burden, with more pronounced effects for mothers (18, 23, 24, 26, 39, 62, 64, 65). However, not all infants have two parents simultaneously available, present, or involved in care. In single-parent or otherwise constrained caregiving contexts, support from extended family, trusted others, or peer networks may become especially important in mitigating burden and supporting participation. In non-therapeutic research, consent rates are higher when both parents are present, suggesting that shared presence may facilitate shared responsibility (65). Extended family and community networks can buffer burden, but may also complicate decisions when value disagreements arise or unverified advice circulates (16, 19, 26, 62). Peer-parent support is often experienced as uniquely helpful in reducing isolation and strengthening coping (16, 29, 40, 63), but should be integrated with clear role boundaries to avoid unintentionally substituting peer emotional support for clinician-provided information and decision support (16, 19, 37).

Taken together, individual- and family-level determinants shape parental involvement by altering parents' capacity to engage with options (cognitive bandwidth), tolerate uncertainty (emotional regulation and meaning-making), and enter structured deliberation (informational readiness and practical feasibility). These mechanisms also point to actionable clinical responses, including treating consent as an iterative process that can be revisited, staging and tiering information over time, and explicitly negotiating roles to accommodate families' changing readiness.

### 5.2 Clinical process-level factors

Clinical process–level determinants translate institutional conditions into concrete “moments of involvement” that parents experience at the bedside. Across studies, the highest-leverage process factors cluster around timing of information, information sources, information delivery and format, and decision aids.

#### 5.2.1 Timing of information

Multiple studies support earlier, staged information provision—for example, an initial consultation at 28–36 weeks' gestation, intensified counseling 5–7 days before an intervention, and a brief recap on the day of the procedure—to allow time for reflection, family discussion, and seeking external information (35, 37–39, 44, 47, 55, 56, 58, 64, 66). When information is delayed until the acute postpartum period, pain, fatigue, and stress may impair decision capacity and increase reliance on default pathways or clinician recommendations (37, 41, 47, 56, 65). Multicenter data suggest that antenatal counseling is associated with calmer and more equitable decision experiences (44). Operationally, these findings support treating consent as a sequence of touchpoints rather than a single event.

#### 5.2.2 Information sources

Parents commonly draw on multiple sources with relatively stable functional roles: standardized booklets/leaflets provide foundational authoritative information; physician teams explain risk; and family members, peers, and online communities often supply experiential and emotional framing (16, 19, 35–37, 39, 42, 44, 48, 50, 55, 63, 66). A process-level implication is therefore to manage—not prohibit—multi-source information use. Teams can direct families to trustworthy materials, ensure consistency across staff, and clarify which questions require clinician input vs. those better addressed through peer experiential support.

#### 5.2.3 Information delivery and format

When feasible, parents prefer face-to-face communication, particularly one-to-one conversations; when bedside presence is not possible, telephone communication is often viewed as essential for maintaining continuity (58, 63, 66). Evidence also suggests that incorporating video into educational materials may improve recall and perceived comprehensibility compared with lengthy written text (36). Across modalities, the central outcome remains whether information is understandable and usable for deliberation. Structured strategies such as Ask–Tell–Ask and teach-back—eliciting parents' prior understanding, explaining risks and benefits in de-jargonized language, and using visual aids to make uncertainty “visible”—can support memory and option comparison (31, 33–36, 39, 40, 50, 62, 63, 66). In pain-related neonatal care, parents report that verbal instruction, concrete recommendations, structured teaching, and bedside demonstration are particularly helpful for enabling participation (24).

#### 5.2.4 Decision aids

When used to supplement rather than replace clinical dialogue, decision aids (written, video-based, or interactive) can yield modest but meaningful benefits, including improved knowledge, greater values–choice concordance, and reduced decisional conflict (31, 42, 43, 52, 56, 67). Randomized trials report small but statistically significant gains in knowledge and material use, with potentially larger effects in some vulnerable populations (56, 67). Effects on choice or consent rates are limited and sometimes slightly negative (56, 67), suggesting that the primary value of decision aids is to strengthen informedness rather than to increase uptake. Notably, these tools are often used infrequently or delivered without structured explanation and without protected time for questions (42). Decision support should therefore be treated as an implementation problem: only when clear touchpoints, scripts, and role accountability are defined can “tool availability” be reliably translated into improved participation quality. For example, one intervention study found that a workflow-embedded decision-support bundle (an infant progress chart paired with prespecified care-planning meetings) reduced maternal uncertainty and unrealistic concerns, lowered decisional conflict, and increased perceived shared decision-making, despite greater baseline illness severity among infants in the intervention group (68).

### 5.3 Team- and organization-level factors

Team- and organization-level determinants influence parental involvement by shaping continuity, role clarity, and the relational conditions under which information is interpreted. Across studies, modifiable factors include team roles and collaboration, physical environment, and time and financial constraints.

#### 5.3.1 Team roles and collaboration

Because bedside nurses are continuously present, use everyday language, and interact frequently, they are often parents' primary source of information, skills coaching, and emotional support (14, 16, 17, 22, 24, 26, 40, 49, 51, 56). In one public-hospital NICU in South Africa, more parents reported that nurses were more helpful than physicians in discussing the infant's condition and treatment (47% vs. 37%) (49). Over time, stable nurse–parent relationships can strengthen parental self-efficacy and willingness to engage through practical guidance and empathic listening (16, 17, 26, 28). Midwives may play a similar early-support role in perinatal care and newborn screening contexts, although impact depends on competence and respectful interaction (28, 37, 58). Physicians typically define the clinical trajectory at key decision points and explain risks and benefits; within family-centered models, their role is to translate complex evidence into actionable options and facilitate explicit values clarification (28, 41, 69). However, nurse availability is not uniformly reassuring: in critical situations, physician unavailability and the delegation of key clinical updates to nurses may be interpreted by parents as insufficient specialist support, thereby undermining trust (22). Trust is built not only through technical competence but also through sustained transparency, responsiveness to questions, and humane communication (16–18, 22, 27, 28, 32, 33, 36, 50, 54, 59, 62). Conversely, within-team disagreement, frequent rotation, and emotional distance can erode trust, leading parents to question whether autonomy is genuine (14, 16–18, 22, 40, 50, 54). A recurring set of conversation-level behaviors supports workable consensus: honest disclosure, proactive invitations to state preferences, empathic responding, explicit acknowledgment of uncertainty, assessment of psychological readiness, and stepwise support for option comparison and values clarification (16–18, 20, 24, 28, 32, 40, 50). From an implementation perspective, these behaviors require clear role delineation (who leads which conversations), coordinated documentation of goals, and alignment on key messages across the team.

#### 5.3.2 Physical environment

Physical space and organizational design determine whether parental presence can translate into meaningful involvement. Noise, interruptions, and open-bay layouts can compress substantive interaction, whereas single-family rooms, overnight accommodation, kangaroo care chairs, and private meeting spaces can increase parental presence and parent–infant contact (24, 51, 62). Survey data suggest that although most parents report feeling welcome, a substantial proportion in open-bay units still feel like “visitors”; in single-room settings, teams more often invite parents to comfort the infant during painful procedures (69). These findings underscore that unit design is not neutral: it shapes whether parents can genuinely enact the parenting role.

#### 5.3.3 Time and financial constraints

Resource constraints shape the time available for deliberation and affect information processing capacity. Financial stress may narrow feasible care pathways and shift risk–benefit trade-offs (26), while fee waivers or free treatment may influence research participation decisions and trust in institutions (39, 54). Work schedules, limited leave, responsibilities for other children, and transportation barriers reduce bedside time and fragment communication into brief encounters, increasing reliance on telephone or video communication (16, 20, 22–24, 29, 32, 37, 39, 40, 55, 57, 63, 64). In decisions about research participation or high-risk interventions, “insufficient time to decide” is commonly cited as a reason for declining, whereas “having enough time” is associated with consent (47, 58, 64). Accordingly, teams may mitigate structural constraints by offering bookable consultation slots, establishing predictable rounding windows, and providing written summaries when parents miss key discussions, thereby converting constraints into manageable process supports.

### 5.4 Policy- and society-level factors

Policy and sociocultural determinants set external boundary conditions for participation before clinician–family interactions begin, by defining parents' rights to be present, access information, and engage in decision-making. Across studies, three domains recur: institutional visitation/contact policies, information governance practices, and broader cultural expectations regarding the “appropriate” parental role.

#### 5.4.1 Policy and institutional context

Visitation restrictions, limits on touch, and insufficient opportunities for skin-to-skin care are repeatedly identified as structural barriers to involvement (22, 24, 26, 28, 40). Technology-dense environments and invasive monitoring may further impede holding and skin-to-skin caregiving, reinforcing parents' sense of constrained roles (22, 24, 28). Although family-centered care frameworks encourage parental participation in daily caregiving tasks, implementation is often bounded by local procedures, and parents frequently experience explicit institutional limits on what they can and cannot do (24, 28). These are not merely differences in preference; they are policy-level determinants that shape the frequency of parent–infant interaction and, in turn, parents' perceived legitimacy to speak in decision conversations.

Information policy also structures participation. A default practice of “no news is good news” may undermine trust in transparency and traceability, particularly when families want systematic feedback of results (37, 58). Some parents call for standardized, predictable procedural guidance at key decision points to provide a reference structure under uncertainty and reduce self-blame during later reflection (15). Together, these findings suggest that transparent and reliable information governance is a prerequisite for sustained involvement, especially when decisions are revisitable.

#### 5.4.2 Sociocultural norms

Broader sociocultural norms influence how parents define what is “normal,” which options are viewed as acceptable, and how responsibility should be allocated. In neonatal circumcision decisions, motivations include aligning the child's identity/status with the father's, religious or cultural practice, and perceived medical/hygiene benefits. In an online survey of 1,235 parents, religious/cultural practice accounted for approximately 32% of stated motivations, whereas medical/hygiene reasons accounted for about 71% (70); another survey reported that approximately 57.4% of parents cited family tradition (27). Beyond shaping the content of parental preferences, cultural norms may also influence parents' preferred decision-making roles. In a cross-cultural study of parents of very low birth weight infants, parents generally preferred an active but not fully autonomous role, and the acceptability of clinician-led, shared, or parent-led decision-making varied across centers (25). These findings suggest that cultural expectations shape not only the starting point for deliberation and the option set that parents perceive, but also how decisional authority and responsibility are understood and distributed. A policy implication is that communication and consent processes should explicitly acknowledge culturally shaped norms while maintaining clear boundaries around voluntariness and the scope of medical recommendations.

## 6 Outcomes of parental involvement in decision-making

Across studies, outcomes are most commonly operationalized in two broad domains: decision-process quality (e.g., perceived SDM, decisional conflict, decisional regret) and emotional outcomes and satisfaction.

### 6.1 Process outcomes

Despite heterogeneity in measurement tools and assessment timing, the evidence shows a consistent pattern: perceived choice and available understanding support are typically associated with lower decisional conflict and decisional regret, and with higher perceived SDM (60, 71). Mechanistic models suggest that perceived choice may exert the strongest direct effect on decision quality, whereas perceived knowledge may operate primarily through indirect pathways (e.g., shaping attitudes and reinforcing the experience of “having a real choice”) (71). This has practical implications for intervention design: increasing information volume alone is unlikely to improve decision quality unless information is structured into actionable options and accompanied by support that enables parents to apply it to deliberation. In research participation and experimental intervention contexts, parents who consent often report lower decisional conflict than those who decline, and this difference remains observable in multivariable analyses (44, 48). The literature also proposes a potential “capacity pathway,” whereby uncertainty reduces self-efficacy, and self-efficacy in turn supports higher perceived SDM (61). Resource factors such as social support and health literacy may influence involvement via mediated pathways; in some models, a substantial proportion of the total effect is attributed to indirect mechanisms (72). Similarly, knowledge may indirectly strengthen involvement by facilitating clearer preference formation, enhancing trust, and improving nurse–parent interaction quality; in some studies, interaction quality appears to be a more influential proximal link (73). At the behavioral level, these process capacities may manifest as better adherence, more timely follow-up, and stronger caregiving capability (36, 74).

Because most evidence is observational and mechanistic models cannot establish causal direction, these associations should be interpreted cautiously. Nevertheless, cross-study consistency supports a reproducible “process triad” as the foundation of high-quality involvement: choice architecture (parents perceive a real, bounded set of options), understanding support (information is usable for trade-offs), and relationship quality (trustful, responsive interaction). These candidate “active ingredients” should be measured alongside downstream psychological outcomes in implementation and evaluation.

### 6.2 Emotional outcomes and satisfaction

Most studies indicate that, in retrospect, parents endorse their decisions and report low overall regret, particularly for screening and other routine pathways. For example, in one genetic screening study, 98.6% of parents considered participation the right decision, 96.7% reported no regret, and 99.3% stated they would make the same choice again (36). In routine newborn bloodspot screening, 99.6% of parents reported willingness to participate again and only 0.5% reported regret (38), with other studies showing similarly high satisfaction and low regret (70). However, mean results may conceal a vulnerable subgroup: after high moral-burden decisions, some parents may experience persistent guilt or distress. One follow-up study reported that approximately 14% of parents attributed ongoing guilt to end-of-life decision experiences, with more pronounced effects among mothers (18). Trust plays a key moderating role: even when involvement is limited in urgent and highly technical decisions, high trust can co-exist with higher satisfaction (33, 43, 54). Relatedly, cross-cultural qualitative evidence suggests that satisfaction does not increase linearly with formal parental autonomy: in units with fewer restrictions on parental visiting and involvement, poor continuity of care was associated with more negative experiences, whereas in more restrictive units characterized by continuous physician availability, consistent messaging, and empathic support, parents reported higher overall satisfaction. This pattern suggests that relational continuity and supportive communication may matter more for emotional outcomes than formal decisional authority (22). Conversely, inconsistent information and lack of opportunities to revisit and renegotiate decisions can increase burden (66). Notably, improvements in decision-process quality do not necessarily translate into higher global satisfaction across all domains: in one intervention study, gains in perceived SDM and decisional conflict were not accompanied by differences in satisfaction with infant care, relationships with clinicians, or satisfaction with treatment decisions (68). Accordingly, emotional outcomes appear to depend less on “which option was chosen” and more on whether the process provides three key conditions: clear communication of uncertainty, preserved space for revisitable discussion, and explicit sharing of moral responsibility. When these conditions are absent, some families may follow a trajectory of sustained emotional burden even when participation appears present on the surface.

## 7 Conclusions and implications

Across neonatal decision contexts, the central finding of this review is that parental involvement should be understood as an adaptive, revisitable longitudinal process rather than a one-time consent moment. Families typically move along a continuum across illness stages—from clinician-led decision-making to SDM, and, in a small subset of high-burden contexts, to parent-led decision-making. Participation quality is generated largely through modifiable communication and workflow design: whether parents perceive a real (and bounded) set of choices, whether information is translated into a structure usable for trade-offs, and whether teams explicitly share moral responsibility under uncertainty.

A practical contribution of this review is the identification of a set of cross-context, implementable “core active ingredients” that can be embedded into routine NICU pathways: (i) choice architecture—naming and delimiting the option set, distinguishing “standard practice” from preference-sensitive choices, and clarifying the boundaries of medically appropriate options; (ii) understanding support—staged and tiered communication paired with teach-back and a concrete comparative structure (benefits, burdens, uncertainty, and time horizons); (iii) relationship quality—proactively inviting questions, acknowledging emotions, and using explicit “shared responsibility” language to prevent SDM from devolving into moral burden shifting. Prior work suggests a perception gap in neonatal decision-making: clinicians may view parental agreement as “making the decision,” whereas parents may experience their role primarily as approving clinician-recommended plans rather than as genuinely sharing decisional authority (75). Accordingly, in practice, parents' roles should be more explicitly defined, and SDM should be made recognizable to parents through visible steps for eliciting and integrating values; and (iv) revisitability—providing protected decision windows and prespecified renegotiation points (e.g., post-crisis debriefs and milestone reviews) to avoid time-critical delegation support hardening into long-term exclusion. Accordingly, the appropriate unit of intervention should shift from “individual parents” to the care system: without clear division of conversational labor and accountable leads, mechanisms for within-team message alignment, and structural supports that make parental presence feasible (visitation policies, private meeting space, predictable rounding/consultation windows, and transparent information governance), SDM is unlikely to be sustained through clinician goodwill alone.

At the research level, the evidence base is constrained by heterogeneity in constructs and measurement time points, and by the predominance of observational designs. Future studies should measure process “active ingredients” alongside downstream outcomes; specify decision context and phase (acute vs. deliberative); test equity-relevant moderators (health literacy, language concordance, and socioeconomic constraints); and use pragmatic designs to evaluate workflow-embedded implementation bundles. Overall, improving parental involvement in the NICU is less about asking parents to “participate more” and more about reliably engineering conditions that make involvement feasible, ethically shared, and psychologically sustainable.
